# Update on Gene Therapy Clinical Trials for Choroideremia and Potential Experimental Therapies

**DOI:** 10.3390/medicina57010064

**Published:** 2021-01-12

**Authors:** Alessandro Abbouda, Filippo Avogaro, Mariya Moosajee, Enzo Maria Vingolo

**Affiliations:** 1 Fiorini Hospital Terracina AUSL, 04019 Terracina, Latina, Italy; a.abbouda@gmail.com; 2 Department of Sense Organs, Faculty of Medicine and Odontology, Sapienza University of Rome, p.le A. Moro 5, 00185 Rome, Italy; filippo@avogaro.org; 3 UCL Institute of Ophthalmology, London EC1V 9EL, UK; m.moosajee@ucl.ac.uk; 4 Moorfields Eye Hospital NHS Foundation Trust, London EC1V 2PD, UK; 5 Great Ormond Street Hospital for Children NHS Foundation Trust, London WC1N 3JH, UK; 6 The Francis Crick Institute, London NW1 1AT, UK

**Keywords:** choroideremia, gene, therapy, clinical trials, stem cells, ataluren, small molecules

## Abstract

*Background and objectives*: Choroideremia (CHM) is an X-linked recessive chorioretinal dystrophy caused by mutations involving the *CHM* gene. Gene therapy has entered late-phase clinical trials, although there have been variable results. This review gives a summary on the outcomes of phase I/II CHM gene therapy trials and describes other potential experimental therapies. *Materials and Methods*: A Medline (National Library of Medicine, Bethesda, MD, USA) search was performed to identify all articles describing gene therapy treatments available for CHM. *Results:* Five phase I/II clinical trials that reported subretinal injection of adeno-associated virus Rab escort protein 1 (AAV2.REP1) vector in CHM patients were included. The Oxford study (NCT01461213) included 14 patients; a median gain of 5.5 ± 6.8 SD (−6 min, 18 max) early treatment diabetic retinopathy study (ETDRS) letters was reported. The Tubingen study (NCT02671539) included six patients; only one patient had an improvement of 17 ETDRS letters. The Alberta study (NCT02077361) enrolled six patients, and it reported a minimal vision change, except for one patient who gained 15 ETDRS letters. Six patients were enrolled in the Miami trial (NCT02553135), which reported a median gain of 2 ± 4 SD (−1 min, 10 max) ETDRS letters. The Philadelphia study (NCT02341807) included 10 patients; best corrected visual acuity (BCVA) returned to baseline in all by one-year follow-up, but one patient had −17 ETDRS letters from baseline. Overall, 40 patients were enrolled in trials, and 34 had 2 years of follow-up, with a median gain of 1.5 ± 7.2 SD (−14 min, 18 max) in ETDRS letters. *Conclusions:* The primary endpoint, BCVA following gene therapy in CHM, showed a marginal improvement with variability between trials. Optimizing surgical technique and pre-, peri-, and post-operative management with immunosuppressants to minimize any adverse ocular inflammatory events could lead to reduced incidence of complications. The ideal therapeutic window needs to be addressed to ensure that the necessary cell types are adequately transduced, minimizing viral toxicity, to prolong long-term transgenic potential. Long-term efficacy will be addressed by ongoing studies.

## 1. Introduction

Choroideremia (CHM) (MIM #303100) is a rare X-linked recessive disorder resulting in progressive degeneration of the photoreceptors, retinal pigment epithelium (RPE), and choroid [1,2]. It has a prevalence of 1 in 50,000–100,000 and is caused by mutations in the CHM gene on chromosome Xq21.2 [3]. It encodes Rab escort protein 1 (REP1), which binds to Rab proteins [4,5], facilitating lipid modification through the addition of a geranyl-geranyl group to their C-terminus (known as prenylation). The prenylated Rab protein is then escorted by REP1 and delivered to the target intracellular compartment [4]. Despite CHM being ubiquitously expressed, the primary site of the disease is the retina, as certain Rabs prefer to be prenylated by REP1 over the isoform REP2 [6].

Due to the X-linked inheritance, males are predominantly affected, demonstrating signs of poor night vision that becomes apparent between the ages of 5 and 25 years [7]. The best corrected visual acuity (BCVA) declines slowly with age, and the mean onset of moderate visual impairment occurs in the fifth decade, and, when the macular involvement is evident, visual acuity (VA) becomes asymmetrical [8]. In some patients with preserved central macula, VA can be maintained until late stages of the disease [7,8,9]. Reduction of VA is associated with patchy peripheral visual field loss, which firstly manifests as a mid-peripheral ring scotoma, with later evolution to the complete loss of the peripheral field [10]. Despite retaining good central VA until advanced stages, early change in color vision is reported [11]. The tritan discrimination is predominantly detected using the Cambridge Colour Test [12]. This defect is easy to explain due to the density reduction of S cones located parafoveally compared to M and L cones located centrally [12,13]. In the early stages, fundus examination shows peripheral pigmentary clumping at the level of the RPE that evolves into areas of chorioretinal atrophy [14]. This degenerative process begins at the equator following a centripetal and centrifugal distribution [15]. The same degenerative process is also noted around the optic disc, while a central island of relatively preserved retinal tissue remains even in advanced stages [16].

In order to investigate these retinal changes, several structural and functional tests have been used [17,18,19,20,21,22,23,24,25]. Fundus autofluorescence (FAF) can monitor the progressive concentric loss of autofluorescence, retaining a residual retinal island at the macula of preserved autofluorescence (PAF) [21]. FAF reflects lipofuscin distribution and the signal originating from the RPE (with the photoreceptors contributing in part) [21,26]. The most common pattern is characterized by decreased FAF with sharp demarcated borders of increased signal from residual degenerating retinal tissue [14,16]. The rate of FAF loss was estimated to be 7.7% per year [21]. Areas of PAF have been reported to be vertically expanded and favoring the central and the temporal side of the macula [20]. Spectral domain optical coherence tomography (SD-OCT) reveals attenuation of the ellipsoid zone, [18,22] reduction of outer nuclear layer thickness [22], and outer retinal tubulations due to primary RPE dysfunction [18,23]. In CHM children until the fourth decade, an asymptomatic increase in central retinal thickness without other signs of retinal edema has been described [16]. In CHM adults, macular cystic edema was also identified [11,18,27] and correlated with progressive decrease in VA and poor prognostic outcomes [18]. OCT angiography (OCTA) [24,28] has shown the ability to detect vascular changes in retinal and choroidal circulations noninvasively in CHM, highlighting decreased vascular density [17,19] that precedes photoreceptor loss [20]. Confocal adaptive optics scanning light ophthalmoscopy (AOSLO) is able to provide effective photoreceptor cellular structure characterization [23,25]. A cone density reduction around the fovea was recognized as the early pathogenic effect of CHM mutation on cellular function [29]. Pathological features were identified as normal foveal cone distribution with peripheral abnormalities or increased foveal cone spacing with normal cone mosaic in retinal eccentricities [23]. Those features are associated with pathological retinal loci and are likely indicative of advanced disease stages [30,31]. Microperimetry highlighted cone and rod system dysfunction sensitivity [21,32,33], with the rod-mediated measurements being more severely affected. Nasal retinal sensitivity appeared to decline earlier than temporal retina, which mirrors a similar pattern of FAF island shrinkage reported previously [21]. Abnormal dark adaptation with a rod intercept time longer than 20 min has been reported [20].

CHM is amenable to gene therapy treatment because it is a monogenic disease, and the cDNA (1.9 kb) is within the size capacity of adeno-associated virus (AAV) vectors (4.7 kb) [34,35]. Lentivirus vectors were used to introduce CHM cDNA in a mouse model, but they showed limited affinity to photoreceptors [36,37]. AAV vectors were able to restore REP1 expression and prenylation function in cell cultures of fibroblasts and lymphocytes derived from CHM patients [38] and improve phagocytosis and trafficking defects in cell cultures of nonhuman primates [39]. Tolmachova and colleagues developed an AAV2 vector (AAV2/2–CBA–REP1) and successfully achieved CHM transgene expression in human and mouse photoreceptors and RPE cells [40]. The advances in preclinical studies lead to the first-in-human gene therapy clinical trial for CHM (NCT01461213) using an adeno-associated virus Rab escort protein 1 (AAV2.REP1) vector in 2011 and from then several multicenter clinical trials worldwide. The aim of this review is to give a summary on the outcomes of CHM gene therapy trials in phase I/II.

## 2. Materials and Methods

Published journal articles based on gene therapy trials for CHM were collected following a specific literature search performed in four stages according to the Preferred Reporting Items for Systematic Reviews and Meta-Analyses (PRISMA) statement [41]. Stage 1 (unique citations): A Medline (National Library of Medicine, Bethesda, MD, USA) search was performed to identify all articles describing gene therapy treatment available in CHM. Keyword searches used were ‘‘CHM’’ and (‘‘AAV + Vectors” or ‘‘gene + therapy + clinical trials’’). Stage 2 (article retrieval): All abstracts from the Medline searches were scrutinized to identify the articles that reported clinical results. Only journal articles published in English were included. Copies of the articles were obtained, and their bibliographies were searched manually for additional articles published in peer-reviewed journals. Stage 3 (article inclusion): Complete articles were reviewed to identify those that reported results of gene therapy in CHM patients [42,43,44,45,46,47,48,49,50,51,52]. Stage 4 (article exclusion): We did not include four clinical trials as the results had not yet been published.

### Data Abstractions and Analysis

A meticulous and systematic review of complete CHM articles was performed. According to the Second Monaciano Symposium [53], clinically meaningful endpoints acceptable to the Food and Drug Administration (FDA) and European Medicines Agency (EMA) and used in CHM clinical trials are reported in Table 1.

In order to compare results among five clinical trials, only BCVA was considered as an outcome because the other parameters were partially reported and not comparable because individual data for each patient were not available. These data are available online at https://www.mdpi.com/1010-660X/57/1/64/s1 Table S1. According to Shen et al. [54], BCVA alone may still have utility as a primary endpoint for patients older than 39 years who have measurable vision decline, while for younger patients additional disease biomarkers are required.

All data were analyzed using Microsoft Excel 97-2003 (Microsoft Corporation, WA, USA). Patient population characteristics were recorded. Complications and their treatments were noted.

## 3. Results

Five CHM gene therapy phase I/II clinical trials were identified for inclusion in this review, and Table 2 summarizes data items from each one [42,43,44,45,46]. The first-in-human gene therapy clinical trial for CHM (NCT01461213) started in 2011 using an AAV2.REP1 vector. In total, 14 patients were recruited at different disease stages and underwent pars plana vitrectomy followed by retinotomy and injection of AAV2.REP1 particles; the median age was 45.5 ± 12.6 SD years (24 min, 72 max). The full report of all 14 cases from this Phase I/II trial, including seven patients who received a high vector dose (1 × 10^11^ gene particles (gp) in 0.1 mL), shows a median gain of 5.5 early treatment diabetic retinopathy study (ETDRS) letters in the treated eye [45]. Median ETDRS baseline letters vs. ETDRS letters at 2 years of follow-up were 68.5 ± 18.8 SD letters (23 min, 89 max) vs. 73 ± 13.9 SD letters (41 min, 94 max), respectively. Overall, the median gain of ETDRS letters was 5.5 ± 6.8 SD (−6 min, 18 max). Complications occurred in two patients due to surgically induced retinal stretching in the first case and intraocular inflammation (vitritis and choroiditis) in the second case [45,55]. Twelve patients without complications gained a median of 5.5 letters above their baseline (ETDRS letters details for each patient are reported in Table 2), while the two patients with complications lost 15 and 14 letters, respectively [45].

Three other clinical trials (in Tubingen, Alberta, and Miami) using the same vector as the NCT01461213 study at the higher dose (1 × 10^11^ gp in 0.1 mL) recruited six patients per trial. In the Tubingen group (NCT02671539) [43], the median age was 52.5 ± 4.1 SD years (51 min, 60 max). Median ETDRS baseline letters vs. ETDRS at 1 year of follow-up were 61 ± 13.2 SD letters (46 min, 77 max) vs. 63 ± 12.5 SD letters (46 min, 77 max), respectively. Only one patient had an improvement of 17 ETDRS letters. In the Alberta group (NCT02077361) [42], the median age was 33.5 ± 5 SD years (29 min, 42 max). Median baseline ETDRS letters vs. 2 years of follow-up was 64 ± 8.7 SD letters (53 min, 75 max) vs. 66 ± 10.1 SD letters (53 min, 76 max), respectively. Overall, a minimal median gain in letters of 0 ± 7.5 SD ETDRS letters (−8 min, 15 max) was reported despite one patient gaining 15 letters and another with a loss of 8 letters in one treated eye (secondary to an intraoperative complication) at 2 years post-treatment. In the Miami group (NCT02553135) [44], the median age was 50 ± 12.7 SD years (32 min, 72 max). Median baseline ETDRS vs. 2 years of follow-up was 63 ± 8.8 SD (56 min, 77 max) vs. 70.5 ± 9.8 (56 min, 79 max), respectively. Overall, there was a median gain of 2 ± 4 SD ETDRS letters (−1 min, 10 max).

The Philadelphia study (NCT02341807) included 10 patients between 26 and 57 years of age at time of injection. Five patients received uniocular subfoveal injections of low dose (up to 5 × 10^10^ gene particles (gp) in 0.1 mL) and five of high dose (up to 1 × 10^11^ gene particles (gp) in 0.1 mL). No data for each individual patient were reported, but by 2 years posttreatment, BCVA returned to baseline in all but one patient who was −17 ETDRS letters from baseline. With the exclusion of this patient, mean BCVA at 2 years was similar in injected (−1.7 letters) compared with uninjected (−0.3 letters) eyes. Two patients showed an improvement of 5–6 ETDRS letters in the injected eye compared with baseline and the uninjected control.

Overall, 40 patients were enrolled. Baseline ETDRS was 65 ± 14.3 letters. Thirty-four patients had a follow-up of 2 years, with a median of 72 ± 11.8 SD (41 min, 94 max) ETDRS letters recorded.

Complications post-injection were reported in 29 of 40 patients. There were two serious adverse events (SAE). One patient had an air bubble in the subretinal space, and one patient had localized foveal thinning after the injection. This, in addition to the adverse event (AE) related to excessive foveal stretch in one patient, resulted in the development of an automated injection system and intraoperative OCT guidance [55,56]. The most common post-injection symptom was metamorphopsia (nine cases), which recovered in eight cases while continuing in one patient. Mild post-operative inflammation was a quite common AE, characterized by anterior chamber reaction (four cases) and vitritis (two cases) that resolved with systemic and topical steroid.

Minor and expected AEs included: subconjuntival hemorrage (12 cases), which resolved in all cases; flashing lights (seven cases), which continued in four cases; and persistent vitreous floaters (two cases). Cataract formation is an expected complication related to the vitrectomy procedure and eight patients had cataract surgery in the treated eye after completion of 2 years follow-up.

Minor and unexpected AEs included changes in intraocular pressure (IOP) level. One patient was diagnosed to have steroid induced ocular hypertension post-injection. The IOP was managed by adding antiglaucoma medications. He recovered with visual field sequelae. Six patients had IOP reduction that resolved spontaneously in all cases. Details about each AE for each clinical trial are reported in Table 3.

There are four ongoing clinical trials in phases II and III. These studies are summarized in Table 4. Up to now, results have not yet been published and hence could not be included for data analysis.

## 4. Discussion

Gene therapy for CHM has reached phase III clinical trials, providing real promise for patients. Review of the ongoing trials has shown that 40 patients have been treated so far with an AAV2-REP1 vector. The most common AEs were subconjunctival hemorrhage, blurred vision metamorphopsia, and a post-operative IOP reduction. The most AE was acute localized foveal thinning, retinal stretching, and intraocular inflammation (vitritis and choroiditis) in three patients. However, overall increased vision with an average gain of 3.1 ETDRS letters (−14 to 18 ETDRS letters) has been ascertained.

Despite the promising results, in order to prolong the long-term transgenic potential and the need for repeat treatments, several challenges remain to be addressed, such as defining the ideal therapeutic window, ensuring that the necessary cell types are adequately transduced, and minimizing viral toxicity. Many of these questions will be answered by ongoing clinical trials, such as the REGENERATE trial phase II (Oxford and Moorfields Eye Hospitals, UK), the GEMINI trial phase II (Tubingen, Germany), and a phase III international multicenter gene therapy STAR trial. Up to now, results have not yet been published for these trials.

Regarding viral toxicity, the vector used for RPE65 retinal dystrophy (Luxturna, Spark Therapeutics Inc., USA) included a strong ubiquitous promoter that targets multiple cell types, including the RPE and the photoreceptors. The solution was adjusted to pH 7.3 and subjected to removal of empty capsids [57,58]. Several strategies are being used to optimize AAV vectors, ranging from the addition of exogenous agents for immune evasion to genetic manipulation of the viral capsid. Continued work in these areas should be followed in order to improve targeting, transgene expression, and immune evasion improving the translational success [59]. The vector construct used, AAV2-CAG-CHM-WPRE-polyA, is identical to the vector used in Luxturna, except for the CHM transgene. In order to reduce post-injection inflammation, all trials used a systemic steroid treatment that included 1 mg/kg/day of prednisolone for 10 days (beginning 2 days prior to gene therapy, on the day of surgery, and for 7 days afterward), followed by 0.5 mg/kg/day for 7 days, 0.25 mg/kg/day for 2 days, and 0.125 mg/kg/day for 2 days. The NCT02671539 (Tubingen) trial also reported a combination of moxifloxacin and dexamethasone eye drops for 21 days. In order to improve the safety profile of the gene therapy and to reduce the risk related to sub-retinal injection, 4D Molecular Therapeutics (4DMT) optimized the AAV vector and designed a new drug: 4D-100 (Roche Pharma AG) comprises an AAV capsid variant carrying a transgene encoding a codon-optimized human CHM gene to be delivered by intravitreal injection. Due to its optimized vector, 4D-110 is a novel gene therapy approach that shows promise in safely treating a broad region of the retina and a broad range of patients. The clinical trial (NCT04483440) was designed to assess the preliminary safety, tolerability, and biological activity of a single intravitreal injection of 4D-110. Up to now, 15 patients were enrolled, and the estimated study completion date is May 2023.

### 4.1. Small Molecule Drugs for CHM

In addition to gene therapy, there are several alternative strategies under development with a potential to treat CHM. About 30% of CHM cases are related to in-frame nonsense mutations, resulting in premature termination codons (PTCs) [60,61]. Small molecule drugs based on aminoglycosides can promote ribosomal read-through of PTCs during translation through competitive binding of near-cognate aminoacyl-tRNAs (tRNAs) instead of eukaryotic release factors (eRFs) [60,62]. In order to halt the progression of recessive disease, 20–25% of wild type levels of functional protein need to be restored [62].

Among the compounds with proven read-through activity are traditional aminoglycosides (gentamicin, paromomycin and geneticin (G418)), the less toxic next-generation designer aminoglycoside-derivatives (NB84, NB74, and NB124), non-aminoglycoside small molecule drugs (PTC124 and PTC414), and small molecule read-through (SMRT) compounds (RTC13, RTC14, GJ071, and GJ072) [62,63]. PTC124 (also known as ataluren or Translarna) has received NICE (National Institute for Health and Care Excellence) approval for Duchenne muscular dystrophy treatment caused by nonsense mutations in the dystrophin gene [64].

In vitro and in vivo preclinical testing of ataluren in models of CHM has led to some promising results with improved REP1 expression [65]. Limitations in the evolution of this treatment are the lack of suitable ocular preparations for targeted drug delivery, the lack of specificity for the gene of interest, hence risk of overriding other random PTCs in the genome, and the decreased availability of transcripts due to nonsense-mediated decay (NMD), thus reducing the substrate for the drug action [62]. It is possible that combining read-through agents with NMD pathway inhibitors (e.g., caffeine, NMDI1, VG1) or dual action agents (amlexanox) could enhance therapeutic benefit [63,66,67,68].

### 4.2. Stem Cell Therapies

Stem cell regenerative approaches are an alternative future treatment for CHM, but there is still a need to identify the correct cell type and the adequate stem cell system in order to achieve tissue regeneration. In addition, the delivery method and the therapeutic window need to be assessed [36].

Skin fibroblast-derived induced pluripotent stem cell (iPSC) technology has been used to differentiate RPE cells, which have been used in clinical trials for treating wet age-related macular degeneration (AMD) in Japan, and retinal progenitor cells were injected intravitreally in a Phase IIb clinical trial for retinitis pigmentosa (NCT02320812) [69]. Recently, hESC-derived RPE cells layered on synthetic membrane have been reported to improve visual acuity (visual acuity gain of 29 and 21 letters, respectively) in two patients with acute wet AMD and rapid deterioration in visual acuity [70]. Further research is required for inherited retinal diseases because, being a chronic disease, they may have long-term structural changes with atrophy that may prevent this form of therapy from being successful.

### 4.3. Neuroprotection Agents

Neuroprotectants are being investigated, such as antioxidants and lutein supplements. They have been found to delay disease progression and result in visual acuity improvement in retinitis pigmentosa [71,72]. Lutein is a xanthophyll carotenoid found in high quantities in green leafy vegetables; it is able to augment macular pigment function through short-wavelength filtration and reactive oxygen species stabilization [73]. Oral supplementation with lutein for 6 months has been studied in CHM patients but there was no measurable benefit in terms of foveal sensitivity and central visual acuity [73].

Reactive oxygen species (ROS) underlie the pathophysiology of diverse neurodegenerative diseases. To control the oxidation process, cells need to activate and deploy endogenous antioxidant defenses. Oxidative stress is caused by an imbalance between the antioxidant defense system and the production of ROS. In the retina, the source and impact of ROS are different depending on the pathology. RPE is particularly susceptible to ROS formation due to its high consumption of oxygen, high proportion of polyunsaturated fatty acids, and constant exposure to light, inducing an increase of photoreceptor cells apoptosis [74]. However, in most inherited photoreceptor degenerations (IPDs), it is suggested that photoreceptors death may be mechanistically different [75]. One possible source of oxidative stress in IPDs is oxygen toxicity in the outer retina due to reduced consumption by photoreceptors mitochondria [76].

Mutations in the glyoxalase 1 (GLO 1) enzyme, involved in the detoxification of a cytotoxic byproduct of glycolysis, were identified in a Sicilian family affected with retinitis pigmentosa (RP). This mutation was suggested to be associated with a faster progression of the retinal disease [77]. At least five other RP causative genes (KLHL7, RDH11, CERKL, AIPL1, and USH1G) suggested a tight connection between induced oxidative stress and RP onset with faster progression [78].

Zebrafish are a well-described CHM model that has been successfully used to highlight the efficient read-through of aminoglycosides and small molecule drugs and their toxic effects. High levels of oxidative stress were associated with *chm*^ru848^ eyes, but once treated with PTC-derived small molecules, the ROS were significantly reduced [65]. Oxidative stress can play a negative role in CHM eyes, and its reduction may be beneficial. Discovering new treatments to counter ROS formation will be a step forward in preventing or slowing down the progression of CHM. Enhancing the production of antioxidant enzymes to reduce ROS or to promote cytoprotective signaling pathways may be a worthy strategy to pursue [79].

### 4.4. Electronic Implants

Electronic retinal implants are an alternative treatment for the final stage of CHM [80]. CHM patients with no light visual perception have been enrolled in several clinical trials testing a 44-channel suprachoriodal bionic eye device (NCT03406416) in Melbourne, Australia, and testing the Intelligent Retinal Implant System (IRIS) V1 (NCT01864486) and V2 (NCT02670980) (Pixium Vision SA). The IRIS II has demonstrated reasonable safety at 6 months with a comparable adverse effect profile compared to the Argus II implant that reaches more than 5 years of follow-up. The IRIS had an increased number of electrodes compared with the Argus II, providing better visual acuity; however, future studies will be needed to further elucidate that result [81].

## 5. Conclusions

This review article outlines the progress made in the field of gene therapy for CHM, and the scientific and clinical community eagerly await the results of the phase III clinical trials. So far, 40 patients have received the AAV2.REP1 vector through subretinal injection. Surgery can be challenging in CHM as there may be a small friable residual retinal island, making delivery of the vector and bleb technically difficult; this is highlighted by the high rate of intraoperative surgical complications, including acute localized foveal thinning, retinal stretching, and intraocular inflammation. Natural history studies have shown that central BCVA remains stable until 39 years [20,54] and that this may not be the most suitable primary outcome measure for clinical trials, but other parameters such as retinal sensitivity and FAF were partially reported and not comparable. There are several other therapeutic strategies under development and so optimal trial design is essential, possibly adopting a multimodal structure–function approach to assess response to treatment. The results of the phase III clinical trials are eagerly awaited to ascertain if gene therapy for CHM will become an approved treatment in the future.

## Figures and Tables

**Table 1:** Functional and structural imaging outcome measures in choroideremia (CHM) clinical trials according to the Second Monaciano Symposium.

Representative Tests	Outcome
BCVA	Visual acuity
Farnsworth D-15	Color vision
Pelli-Robson; quick CSF	Contrast sensitivity
Microperimetry	Macular sensitivity
Questionnaires: VFQ-25, Cardiff Visual Ability	Patient-reported visual outcome
Speed; Precision	Reading
SD-OCT	Retinal structure
FAF	Autofluorescence

Legend: BCVA: best corrected visual acuity; CSF: contrast sensitivity function; VFQ: Visual Function Questionnaire; SD-OCT: spectral domain optical coherence tomography; FAF: fundus autofluorescence.

**Table 2:** Completed and registered studies involving CHM gene therapy with AAV2-REP1.

Clinical Trial Registration, Location and Phase	Patient	Age	BCVA Baseline ETDRS	BCVA 1 Year	BCVA 2 Years	Mutation
NCT01461213 University of Oxford, UK October 2011 Sponsor: University of Oxford Phase I/II low and high dose, open label, 14 male participants, subretinal injection AAV2-REP1 (AAV2-CAG-CHM-WPRE-polyA)	L1	63	23	45	41	c.940+2T>C
	L2	47	79	79	73	c.189+1G>C
	L3	36	89	91	94	c.492_493delGA
	L4	55	53	69	61	c.535_538delGAAA
	L5	41	79	75	76	c.529delG
	H1	38	77	78	78	c.799C>T
	H2	43	76	74	80	c.877C>T
	H3	41	70	70	79	c.1264C>T
	H4	59	61	61	70	c.1335_1336insA
	H5	72	67	70	73	c.757C>T
	H6	55	60	60	63	c.799C>T
	H7	24	39	54	53	c.525_526delAG
	#C1	57				c.819+1G>T
	#C2	44				c.130G>T
NCT02077361 University of Alberta, Canada April 2015 Sponsor: Ian M. MacDonald Phase I/II single dose, open label, 6 male participants, subretinal injection AAV2-REP1 (AAV2-CAG-CHM-WPRE-polyA)	P1	42	53	No data available for the 1 year follow-up	53	c.315-?_1166+?del
	P2	35	61		76	c.1218C>A
	P3	29	67		59	c.1218C>A
	P4	38	73		73	c1245-521A>G
	P5	32	58		58	c.224G>A
	P6	30	75		75	c1245-521A>G
NCT02553135 University of Miami, USA September 2015 Sponsor: Byron Lam Phase II single dose, open label, 6 male participants, subretinal injection AAV2-REP1 (AAV2-CAG CHM-WPRE-polyA)	501	50	65	69	70	Arg450MetAG(G)>AT(G)
	502	53	61	71	71	c.525_526delAG
	503	49	56	54	56	Arg239StopCGA>TGA
	504	72	58	58	57	c.525_526delAG
	505	50	75	78	77	Thr175del2acAG
	506	32	77	80	79	p.Arg267Ter(R267X)
NCT02671539 University of Tubingen, Germany January 2016 Sponsor: STZ eyetrial Phase II single dose, open label, 6 male participants, subretinal injection AAV2-REP1 (AAV2-CAG-CHM-WPRE-polyA)	401	52	75	71	Average change of 3.7 ± 7.5 ETDRS letters(min −4.2, 11.6 max)	c.1467delA
	402	60	46	46		c.887C>T; p.R293X
	403	53	47	64		Exon 1-15_del
	404	59	61	47		c.757C>T
	405	51	77	77		c.800delT (pT288Lfs*3)
	406	51	61	62		p.S218X
NCT02341807 * Children’s Hospital of Philadelphia, University of Pennsylvania, Massachusetts Eye and Ear Infirmary January 2015 Sponsor: Spark Therapeutics Phase I/II safety study in subjects with CHM gene mutations using an AAV2-REP1 (AAV2-CAG-CHM-WPRE-polyA)	10 adult subjects		BCVA unchanged among 2 years of follow-up in 9/10 subjects, one patient lost 17 ETDRS letters			Data not available

* Single data for each patient was not available. # These two patients had severe post-injection complications, and they were excluded from the trial. No data are available. Legend: AAV2.REP1 adeno-associated virus Rab escort protein 1 vector; BCVA: best corrected visual acuity; ETDRS Early Treatment Diabetic Retinopathy Study.

**Table 3:** Main post-injection ocular complications reported for each trial *.

Trial	Complication	Number of Cases
NCT01461213 University of Oxford, UK October 2011	Flashing lights	7/14
	Slight ocular discomfort post-op	5/14
	Blurred vision	4/14
	Metamorphopsia	3/14
	Colours appear “washed out”	3/14
	Development of cataract	5/14
	Foreign body sensation	2/14
	Slightly painful eye	1/14
	Violet coloured tint to vision	1/14
	Micropsia	1/14
	Raised intraocular pressure	1/14
	Vision feel “duller”	1/14
	Vitritis	1/14
	Mild post-op inflammation	1/14
	Excessive foveal stretch	1/14
	Air bubble in balanced salt solution (BSS) injection system during surgery	1/14
	Subconjunctival hemorrhage	1/14
	Suture-related conjunctival inflammation	1/14
NCT02077361 University of Alberta, Canada April 2015	Subconjunctival hemorrhage	6/6
	Intraocular pressure (IOP) decrease post-op	6/6
	Blurred vision	6/6
	Metamorphopsia	6/6
	Ocular pain	4/6
	Vitreous floaters	2/6
	Anterior chamber reaction	2/6
	Intra-retinal hyperreflective material	1/6
NCT02553135 University of Miami, USA September 2015	Conjunctiva hemorrhage, edema	6/6
	Subretinal fluid	5/6
	Extrafoveal macular retinal hole in area of nonfunctioning retina	2/6
	Anterior chamber cells	2/6
	Vitreous cells	1/6
	Diplopia	1/6
	Worsening of pre-existing cataract	1/6
NCT02341807 Children’s Hospital of Philadelphia, University of Pennsylvania, Massachusetts Eye and Ear Infirmary January 2015	Acute (~72 h) localized foveal thinning after injection	1/10

* Complications related to NCT02671539 trial are not available.

**Table 4:** Active and uncompleted studies involving choroideremia treatment with AAV2-REP1.

Clinical Trial (clinicaltrials.gov)	Registration	Phase and Study Type
NCT02407678	University of Oxford and Moorfields Eye Hospital, UK REGENERATE TRIAL August 2016	Phase II Randomised, single dose, open label, 30 male participants, subretinal injection AAV2-REP1 (AAV2-CAG-CHM-WPRE-polyA)
NCT03507686	Nightstar Therapeutics, international, multicenter GEMINI TRIAL November 2017	Phase II single dose, open label, two-period, 15 male participants, bilateral subretinal injection AAV2-REP1 (AAV2-CAG-CHM-WPRE-polyA)
NCT03496012	Nightstar Therapeutics, international, multicenter STAR TRIAL December 2017	Phase III control, low and high dose, randomised, open label, outcomes-assessor masked, prospective, parallel controlled group study, 140 male participants, subretinal injection AAV2-REP1 (AAV2-CAG-CHM-WPRE-polyA)
NCT04483440	4D Molecular Therapeutics, US July 2020	Phase I open-label, dose-escalation study of the safety, tolerability, and preliminary efficacy of intravitreal 4D-110

## Data Availability

No new data were created in this study. Data sharing is not applicable to this article.

## Supplementary Materials

The following are available online at https://www.mdpi.com/1010-660X/57/1/64/s1, Table S1: Additional data available for each trial.
