# Supplementary material for: Update on Gene Therapy Clinical Trials for Choroideremia and Potential Experimental Therapies
Source: Medicina (Kaunas). 2021 Jan 12;57(1):64. doi: 10.3390/medicina57010064 (PMC7826687; doi:10.3390/medicina57010064)
Supplement: Supplementary file 1 [file medicina-57-00064-s001.pdf]

Table S1. Additional data available for each trial.

Clinical trial registration (clinicaltrial.gov)	Patient	Age	Mean sensivity (dB) baseline	Mean sensivity (dB) after 6 months
NCT01461213 University of Oxford, UK October 2011	P1	63	23	28
	P2	47	25	27
	P3	35	24	28
	P4	57	23	23
	P5	41	18	20
	P6	56	25	26
	**Patient**	**Age**	**FAF (mm2) baseline**	**FAF (mm2) after 2 years**
	L1	63	1.32	1.25
	L2	47	4.16	3.12
	L3	36	9.96	8.77
	L4	55	1.74	1.48
	L5	41	2.71	2.00
	H1	38	11.95	7.75
	H2	43	3.47	2.71
	H3	41	6.43	5.59
	H4	59	3.73	3.30
	H5	72	2.56	2.43
	H6	55	0.82	0.54
	H7	24	1.28	0.93
NCT02553135 University of Miami, USA September 2015	**Patient**	**Age**	**Mean sensivity (dB) baseline**	**Mean sensivity (dB) after 2 years**
	501	50	1,9	0,8
	502	53	0	4,1
	503	49	0	0
	504	72	0	0,2
	505	50	0	0
	506	32	5.1	5.7
NCT02671539 University of Tubingen, Germany January 2016	**Patient**	**Age**	**FAF (mm2) baseline**	**FAF (mm2) after 1 year**
	401	52	10.01	8.41
	402	60	2.47	2.11
	403	53	3.36	2.82

	404	59	1.00	0.77
	405	51	9.67	8.44
	406	51	16.66	14.8
	**Patients**	**Median age**	**Mean sensivity (dB) baseline mean ±SD**	**Mean sensivity (dB) change after 2 years mean ±SD**
	5 subjects	52.5 ± 4.1 SD	9.8±5.0 (min 3.6, 16 max)	0.6±7.1 (min -8.3,max 9.4)
**NCT02341807* Children's Hospital of Philadelphia, University of Pennsylvania, Massachusetts Eye and Ear Infirmary January 2015**	**Patients**	**Age**	**Mean sensivity (dB) after 2 years mean ±SD**	
	10 subjects	Between 26 and 57 years old	0.7±0.75	

NCT02077361 trial was not included because it did not provide any additional data
*Only cumulative data available for NCT02341807 trial
